# Alterations of biomarker profiles after neoadjuvant chemotherapy in breast cancer: tumor heterogeneity should be taken into consideration

**DOI:** 10.18632/oncotarget.5050

**Published:** 2015-09-05

**Authors:** Xingchen Zhou, Junyong Zhang, Haiqin Yun, Ranran Shi, Yan Wang, Wei Wang, Svetlana Bajalica Lagercrantz, Kun Mu

**Affiliations:** ^1^ Department of Pathology, Shandong University School of Medicine, Jinan 250012, China; ^2^ Department of Gastroenterology, Provincial Hospital Affiliated to Shandong University, Jinan 250021, China; ^3^ Department of Hematopathology, The University of Texas MD Anderson Cancer Center, Houston, Texas 77030, USA; ^4^ Department of Oncology-Pathology, Karolinska Institutet, CCK Karolinska University Hospital, Stockholm SE-171 76, Sweden

**Keywords:** breast cancer, neoadjuvant chemotherapy, biomarkers, heterogeneity

## Abstract

Tumor biomarkers including estrogen receptor (ER), progesterone receptor (PR), human epidermal growth factor receptor 2 (HER2) and Ki-67 are routinely tested in breast cancer patients and their status guides clinical management and predicts prognosis. A few retrospective studies have suggested that neoadjuvant chemotherapy (NAC) in breast cancer may change the status of biomarker expression, which in turn will affect further management of these patients. In this study we take advantage of a relatively large cohort and aim to study the effect of NAC on biomarker expression and explore the impact of tumor size and lymph node involvement on biomarker status changes. We collected 107 patients with invasive breast cancer who received at least three cycles of NAC. We retrospectively performed and scored the immunohistochemistry (IHC) of ER, PR, HER2 and Ki-67 using both the diagnostic core biopsies before NAC and excisional specimens following NAC. HER2 gene status was assessed by fluorescence in situ hybridization for cases with IHC result of 2+. We demonstrated that there was a significant decrease in expression of PR (*P* = 0.013) and Ki-67 (*P* = 0.000) in post-NAC specimens compared to pre-NAC core biopsies. In addition, cases with large tumor size (≥2cm) and cases with lymph node metastasis were more frequently to have biomarker changes. Finally we studied cases with HER2 status changes after NAC treatments in detail and emphasized the nature of tumor heterogeneity.

## INTRODUCTION

Breast cancer is the most commonly diagnosed cancer in women and is the leading cause of cancer-related death amongst women worldwide. Chemotherapy is an important treatment modality and has significantly improved the survival of breast cancer patients. In chemotherapy, neoadjuvant chemotherapy (NAC) has become a well-established approach to treat large-sized or locally advanced breast cancer [1–3]. The main purpose of NAC is to shrink tumor size, improve the chance of surgical operation, and monitor tumor response to chemotherapeutic agents. Its clinical value for predicting pathologic complete response (pCR) and its effect on tumor biology and biomarker expression, however, are not fully understood [4, 5].

The measurement of ER, PR, HER2 and Ki67 by IHC has become the standard practice in clinical managements of breast cancer patients. Their expression pattern has been used clinically to guide therapy and predict survival. They are also used as surrogate markers for subtype classification in breast cancer. Retrospective studies have suggested that the expression status of ER, PR, HER2 and Ki-67 may differ between the initial diagnostic core biopsies and excisional specimens after NAC [6, 7]. The changes of these biomarkers have clinical significance as oncologists need to make appropriate treatment adjustment according to the status of these biomarkers. Thus a comparison of biomarker changes before and after NAC treatment is helpful for further clinical management and is also useful for studying tumor biological behavior [8].

The mechanisms that mediate biomarker status change in breast cancer are not fully understood and several potential explanations are present. The initial core biopsy for diagnosis is usually small in size and may mix with non-tumoral tissue, interfering the interpretation of IHC results. Second, tumor heterogeneity may play a role in the discordance between core biopsy and excisional specimens as the initial core biopsy may not be representative of the whole tumor. Finally, the biomarker status change may be caused by NAC, which selectively kill some sensitive tumor cells and leave resistant clones behind.

Although the effect of NAC on biomarker expression has been studied, the results were controversial. While some studies have shown that NAC alters biomarker expression, others did not show such effect [9]. In addition, there is no study to investigate the relationship between biomarker changes and various tumor parameters, such as tumor size and nodal involvement. Finally although tumor heterogeneity has been proposed as a potential mechanism of biomarker change after NAC, there is no study that elucidated this in detail. In this study, we aimed to explore the NAC induced biomarker changes in a more precise way. We evaluated the frequency of status changes in each biomarker before and after NAC treatment. We also investigated the impact of tumor size and the status of lymph node on biomarker status change. In addition, we studied five cases with HER2 discordance before and after NAC treatment and aimed to emphasize the heterogeneity nature of breast cancer.

## RESULTS

### The comparison of biomarker expression in core biopsies and post-NAC excisional specimens

**Figure 1:**
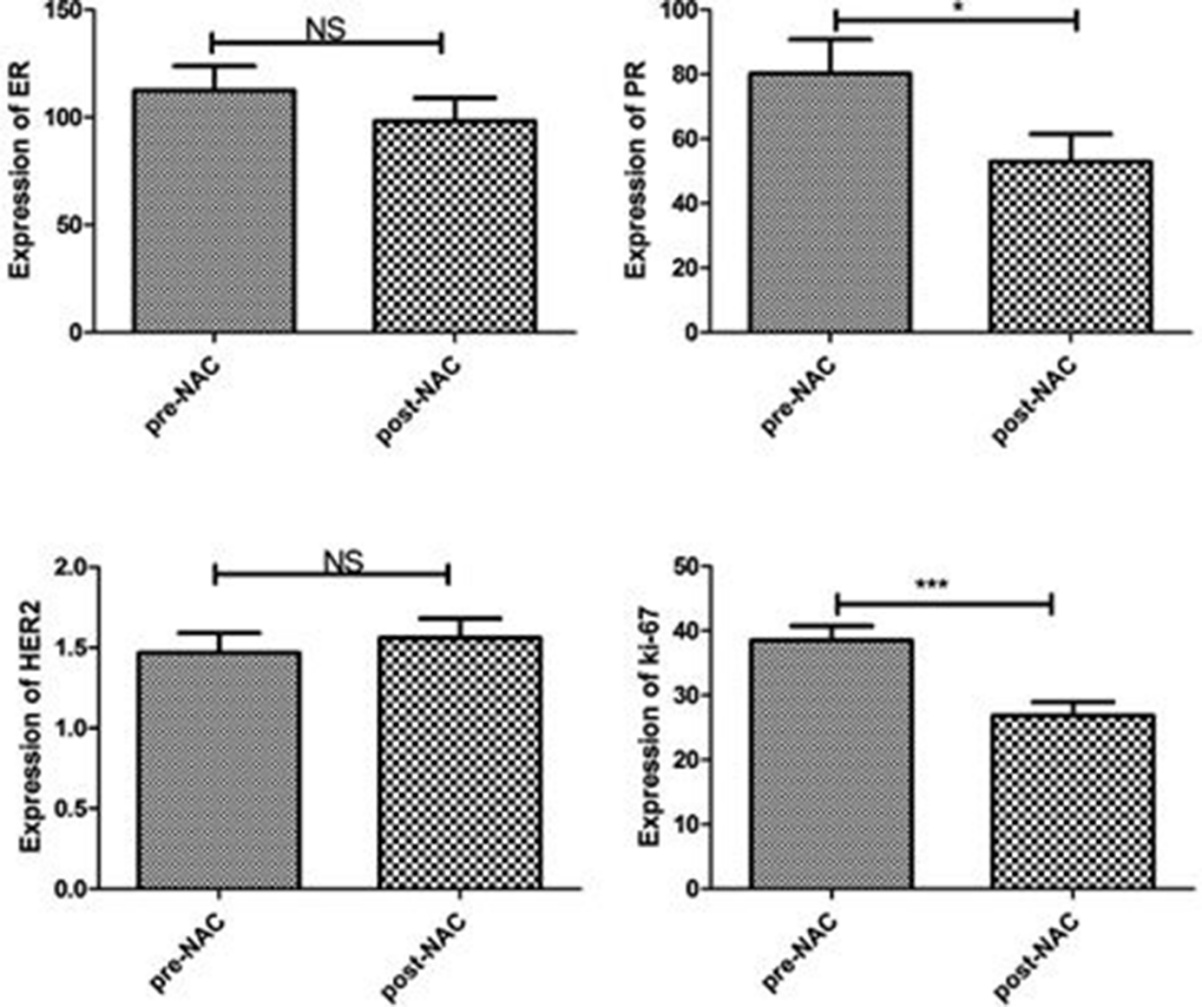
Paired analysis of biomarker changes after NAC PR expression and Ki-67 index were significantly decreased in post-NAC excisional specimens when compared to pre-NAC core biopsies (*P < 0.05, ***P < 0.001, NS: No significant difference).

We retrospectively scored the IHC of ER, PR, HER2 and Ki-67 in core biopsies and surgical excisional specimens of 107 breast cancer patients. Their clinicopathological features are shown in Table 1. ER and PR status was assessed following both H-score scoring method [10] and the guidelines from American Society of Clinical Oncology (ASCO) and College of American Pathologists(CAP) [11]. Among 107 NAC-treated cases, changes of ER, PR, and HER2status were observed in 14%(15/107), 24.3%(26/107), and 4.7% (5/107) cases respectively. We further compared the ER and PR expression level between core biopsies and post-NAC excisional specimens using H-score scoring semi-quantitative method. A significant reduction of PR expression (*P* = 0.013) was found in post-NAC tumors when compared to the pre-NAC core specimens, while no significant change was found for ER (*P* = 0.100) and HER2 (*P* = 0.239) (Figure 1). By quantitative counting of proliferative marker Ki-67, we found a significantly reduced expression in post-NAC specimens (*P* = 0.000) (Figure 1).

**Table 1:** Clinicopathological features of 107 core biopsy breast carcinomas before NAC

	*N*	(%)
Age		
<40	20	18.7%
≥40	87	81.3%
Biomarker status		
ER+	73	68.2%
ER-	34	31.8%
PR+	53	49.5%
PR-	54	50.5%
HER2+	42	39.3%
HER2−	65	60.7%
Ki-67 < 14%	17	15.9%
Ki-67 ≥ 14%	90	84.1%
NAC		
AT or ET	51	47.7%
CAF or CEF	17	15.9%
CAT or CET	17	15.9%
others	22	20.6%

ER = estrogen receptor; PR = progesterone receptor; HER2 = human epidermal growth factor receptor-2; A = Adriamycin; T = Taxotere; E = Epirubicin; C = cyclophosphamide; F = 5-fluorouracil.

The tumors were further classified into luminal A, luminal B, HER2 amplified, triple negative, and luminal-HER2 groups. A detailed comparison of biomarker status changes in different molecular subtypes is summarized in Table 2. Notably, no receptor status alternation was observed in the triple negative group. Next we examined the change of Ki-67 in different molecular subtypes. The luminal B as well as Luminal-HER2 tumors showed a significant reduction in Ki-67 expression after NAC while no significant difference was found in the luminal A, HER2 amplified or the triple negative tumors (Table 3). There was no significant difference in ER, PR and HER2 expression before and after NAC treatment in all subtypes (Supplementary Table S1).

**Table 2:** Biomarker profiling changes after NAC treatment with regard to molecular subtypes in breast cancer

		ER			PR			HER2		
Molecular subtype	*N*	No change	+ → −	− → +	No change	+ → −	− → +	No change	+ → −	− → +
Luminal A	14	12(85.7%)	2	0	11(78.6%)	2	1	13(92.9%)	0	1
Luminal B	38	33(86.8%)	4	1	26(68.4%)	6	6	37(97.4%)	0	1
HER2 amplification	21	18(85.7%)	0	3	18(85.7%)	0	3	21(100%)	0	0
Triple negative	13	13(100%)	0	0	13(100%)	0	0	13(100%)	0	0
Luminal-HER2	21	16(76.2%)	5	0	13(61.9%)	5	3	18(85.7%)	3	0
Total	107	92(86%)	11	4	81(75.7%)	13	13	102(95.3%)	3	2

**Table 3:** The change of Ki-67 expression after NAC in different molecular subtypes of breast cancer

		Ki-67 (mean ± SE)		
Molecular subtype	*N*	pre-NAC	post-NAC	*P*
Luminal A	14	8.64 ± 0.77	11 ± 3.19	0.649
Luminal B	38	36.05 ± 3.29	21 ± 3.17	0.001*
HER2 amplification	21	40.95 ± 4.25	35.00 ± 4.18	0.221
Triple negative	13	65.38 ± 6.73	47.92 ± 7.81	0.099
Luminal-HER2	21	43.81 ± 15.88	26.81 ± 3.88	0.002*

Wilcoxon test for paired data was used to compare biomarker changes after NAC.

* *P* < 0.05 was considered statistically significant.

### Biomarker changes and clinicopathological parameters

The 107 breast carcinomas were further stratified by tumor size, axillary lymph node status and chemotherapy response (Miller and Payne (MP) grading system). A significant decrease in PR and ER expression was found in tumors greater than 2cm, but not in tumors with smaller size. In terms of lymph node status, we found a significant reduction of PR expression in tumors with positive lymph node involvement, while no difference was found in cases with negative lymph node involvement. No significant changes were found for HER2 status among all groups of the tumors. Ki-67 was significantly decreased after NAC in all groups except tumors without axillary node metastasis. Table 4 summarized biomarker changes in post-NAC excisional specimens in relation to various clinicopathological parameters.

**Table 4:** The change of ER, PR, and Ki-67 in relation to clinicopathological parameters

	ER (mean ± SE)			*P*	PR (mean ± SE)		*P*	Ki-67 (mean ± SE)		*P*
	*N*	pre-NAC	Post-NAC		pre-NAC	Post-NAC		pre-NAC	Post-NAC	
Tumor size										
<2 cm	31	83.06 ± 19.43	101.61 ± 21.95	0.426	70.68 ± 18.49	55.81 ± 16.94	0.272	48.71 ± 4.53	33.00 ± 4.41	0.001*
≥2 cm	76	124.57 ± 13.75	96.99 ± 12.20	0.016*	84.26 ± 12.79	51.78 ± 10.01	0.029*	34.36 ± 2.45	24.34 ± 2.35	0.002*
Node status										
-	22	93.05 ± 25.23	96.27 ± 23.95	0.851	66.41 ± 22.81	40.91 ± 18.89	0.214	47.73 ± 5.10	39.36 ± 5.96	0.135
+	85	117.59 ± 12.76	98.86 ± 12.02	0.088	83.93 ± 11.88	56.06 ± 9.68	0.031*	36.13 ± 2.47	23.61 ± 2.06	0.000*
Chemo-response										
CR	54	100.65 ± 16.06	83.72 ± 15.03	0.137	79.39 ± 15.21	52.83 ± 12.71	0.061	38.85 ± 3.31	29.19 ± 3.07	0.023*
CS	53	124.66 ± 16.09	113.21 ± 15.09	0.338	81.28 ± 14.66	53.06 ± 11.68	0.098	38.17 ± 3.10	24.46 ± 2.93	0.000*

Wilcoxon test for paired data was used to compare biomarker changes after NAC.

* *P* < 0.05 was considered statistically significant.

CR: chemotherapy-resistant, CS: chemotherapy-sensitive.

We further divided breast carcinomas into chemotherapy-resistant (CS) and chemotherapy-sensitive (CR) groups according to MP grading system on the excisional specimens (detail in material and methods). We found a significant reduction in Ki-67expression in both CS and CR groups (Table 4). No significant alterations were found for ER, PR and HER2 expression.

### Case study of HER2 intra-tumor heterogeneity

Next we focus on cases with HER2 status alterations after NAC. There were five cases with discordant results between initial biopsies and the following excisional specimens. HER2 expression changed from negative to positive in two cases and from positive to negative in three cases (Supplementary Table 2).

For two cases with HER2 from negative to positive, both post-NAC samples showed low level of HER2 amplification with cell to cell variation. One case showed a combination of three tumor components in the excisional specimen: invasive ductal carcinoma, NOS, invasive lobular carcinoma, and invasive micropapillary carcinoma. The core biopsy was only composed of HER2 negative invasive lobular carcinoma.

**Figure 2:**
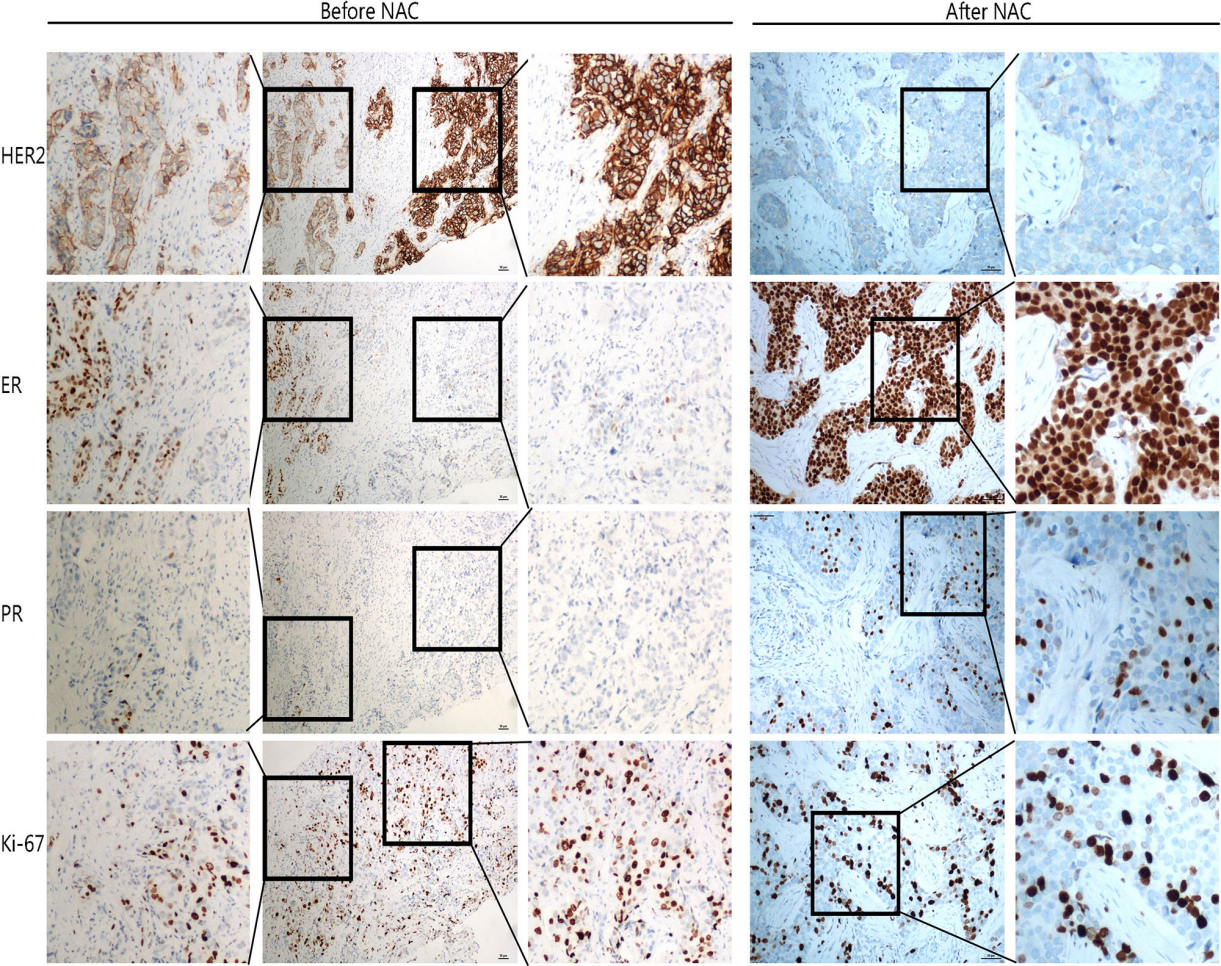
IHC of case QL25 demonstrated tumor heterogeneity and altered biomarker expression patterns after NAC treatment In the core biopsy (left), there are areas with HER2 IHC2+ and adjacent areas with IHC3+. In the IHC2+ regions, ER positivity was moderate, along with weak PR expression and relatively low Ki-67 index (25%); in the IHC3+ areas, ER positivity was weak, PR was negative, and Ki-67 index was 45%. After NAC (right), HER2 was changed to 1+, along with strong ER expression and moderate PR positivity. The Ki67 index was 35%.

**Figure 3:**
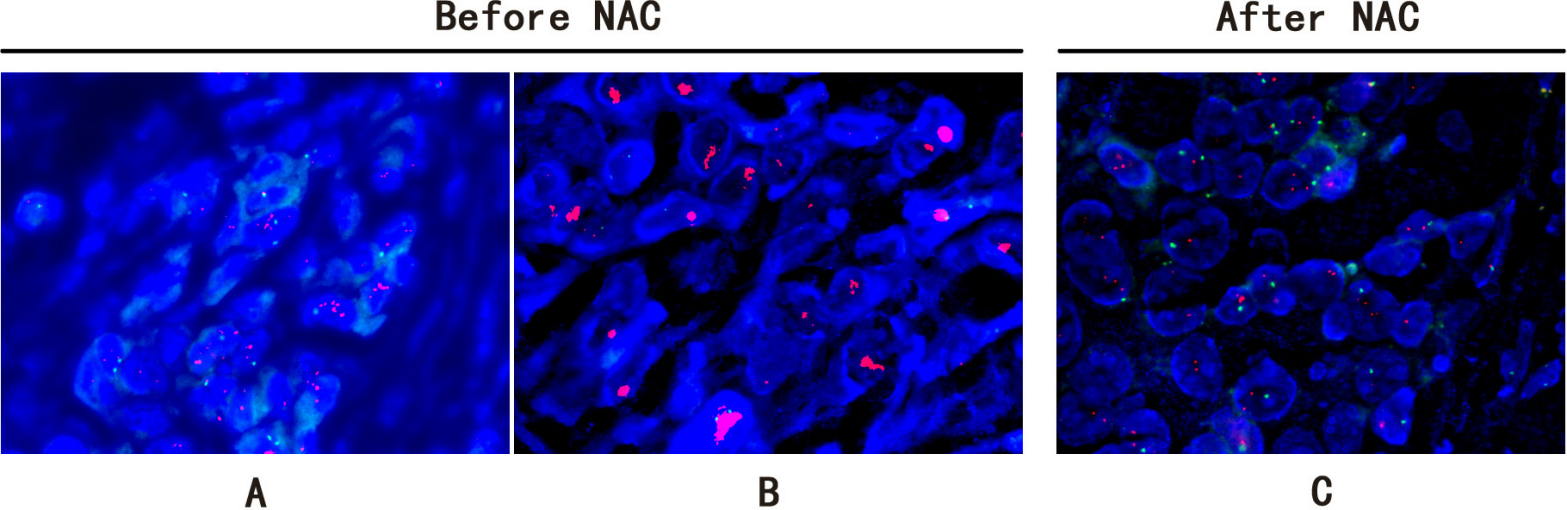
FISH study of breast carcinoma from case QL25 showing HER2 heterogeneity and status change after NAC treatment FISH demonstrated obvious tumor heterogeneity with HER2 dot amplification (HER2/CEP17 ratio = 3) **A.** and adjacent cluster amplification **B.** in pre-NAC core biopsy. After NAC treatment, HER2 was changed to negative (HER2/CEP17 ratio = 1.41) **C.**

Of three cases with HER2 status from positive to negative, two had HER2 protein of 2+ before NAC. FISH test confirmed HER2 gene amplification at a low level with cell to cell heterogeneity. For the third case, HER2 protein was altered from IHC3+ (positive) to IHC1+ (negative). FISH confirmed HER2 cluster amplification in pre-NAC core biopsy, and negative HER2 (HER2/CEP17 ratio = 1.41) after treatment. As the genetic loss of amplified HER2 after NAC treatment is rare, we therefore studied the morphology as well as the IHC of this case in detail. IHC showed variable HER2 expression with 3+ in some areas and 2+ in other areas (Figure 2). This was confirmed by FISH which also showed obvious tumor heterogeneity with HER2 gene cluster amplification in the IHC 3+ areas, and dot amplification (HER2/CEP17 ratio = 3) in the IHC2+ components (Figure 3). Both ER and PR protein expression was weaker in the HER2 3+ areas than HER2 2+ areas. After NAC, the IHC of HER2 was changed to 1+, along with increased ER and PR expression (Figure 2).

## DISCUSSION

NAC is frequently utilized to treat patients with locally advanced breast cancer. Assessment of biomarker changes after NAC treatment is critical for evaluation of NAC efficiency and should be used to tailor the clinical management for breast cancer patients. Changes of biomarker status in breast cancer after NAC treatment have been described in the literature [6, 12]. The mechanisms mediating the changes of biomarker expression, however, remain largely unknown. Whether these changes are due to NAC treatment, tumor heterogeneity, sampling or technical issues need to be clarified.

In the current study, we demonstrated that the biomarker expression changed in a significant proportion of patients who received NAC. Among ER, PR and HER2, PR was most frequently altered, occurring in 24% of cases after NAC. Our findings are consistent with other previous reports [13, 14]. In this study we also evaluate the relationship between PR loss and MP grade and we found that there was no significant correlation between PR loss and MP grade. The significance of biomarker alternation in breast cancer has been studied in few studies. In a prospective study of 423 breast cancer patients by Jin et al., alteration in hormone receptor status after NAC was an independent prognostic factor for worse disease-free survival (DFS) and overall survival (OS) [15]. It is well known that ER+/PR− tumors had a higher level of growth signaling than ER+/PR+ tumors [16]. PR loss may reflect a relatively poor response to chemotherapy and may be associated with a poor prognosis [17, 18].

According to gene expression profiling and immunophenotypic pattern, breast cancer can be further classified into different biologic subtypes: luminal A (ER+/PR+/HER2−, Ki67 < 14%), luminal B (ER+/PR+/HER2−, Ki67 ≥ 14%), luminal-HER2 (ER+/PR+/HER2+), HER2+ (ER−/PR−/HER2+), and triple negative (ER−/PR−/HER2−). These subtypes are associated with different clinical outcomes. In our study, we analyzed biomarker changes in different subgroups. None of the studied biomarkers were changed in the triple negative group of tumors, while luminal-HER2 subgroup showed the most frequently altered biomarker status after NAC treatment (Table 2).

Besides the classification of breast cancer into five groups based on gene expression profiling as described above, recent studies took a different approach and classify breast cancer based on genomic alterations. Three genetic categories were identified; “simplex” type characterized by a few genomic rearrangements; “complex sawtooth” characterized by more rearrangements and gene copy number alterations; “complex firestorm” characterized by high intensity of gene amplification profiles limited to single chromosome arms [19]. Luminal A type correlates with the simplex profile such as gain of 1q and loss of 16q [20]. In luminal A tumors, the frequency of ER and PR positive cells are nearly 100% and their expression is homogeneously strong. A large portion of IHC HER2 3+ tumors showed HER2 gene cluster amplification by FISH. This type of breast tumor is probably related to “complex firestorm” profile. For luminal-HER2 subtype, we believe this type of tumor might be more heterogeneous and is related to “complex sawtooth” profile, which correlates with genomic instability and the potential to bypass the chemotherapy treatment. Consistently we found this subtype is most commonly to have biomarker changes (Table 2).

In this study, we also assessed whether there is a relationship between biomarker changes and tumor parameters, such as tumor size and lymph node status. We found a decreased PR and ER expression in tumors with large size (>2 cm) (Table 4). In addition, tumors with nodal involvement were more frequent to have decreased PR expression (Table 4). The rationale behind these findings is at least partially attributable to the tumor heterogeneity. It is plausible to state that large tumors are more variable from areas to areas and thus the initial core biopsy specimen is less likely to represent the whole tumor. Also as tumor grows and metastasizes to lymph nodes, microenvironment change and clonal evolution occur, which enhance the tumor heterogeneity.

The breast cancer heterogeneity is clearly demonstrated in our study (Figure 2 & 3). Of note, two specific aspects of tumor heterogeneity should be emphasized: morphological heterogeneity and molecular profiling heterogeneity. It is obviously easy to recognize morphological heterogeneity, such as a breast cancer composed of invasive ductal carcinoma and micro-papillary carcinoma. But for the heterogeneity at molecular level, it is much more difficult to detect and currently there is no standard guideline to measure and report molecular heterogeneity. In our study, for the case with HER2 from 3+ to 1+ after NAC, there was no heterogeneity at morphological level. However, at molecular level, the tumor was heterogeneous. As shown in Figure 2, more than 50% of tumors cells showed HER2 staining of 3+, along with reduced ER and PR expression, while the rest of the tumor showed moderate HER2 protein expression (2+) accompanied with higher expression of ER and PR. FISH result further confirmed HER2 gene cluster amplification in IHC 3+ areas, and dot amplification in IHC 2+ tumor components (Figure 3). So for this case, it is a heterogeneous tumor composed of two subtypes; HER2 amplification subtype and luminal-HER2 subtype. Currently there is no standardized therapy for breast cancers with molecular heterogeneity, but it is plausible to propose that this kind of breast cancer should be treated differently and combined therapeutic regimens may be needed. Of note, the current IHC reporting system for HER2 and hormone receptor is based on overall expression of the whole tumor. In our study, we demonstrated tumor heterogeneity is present in some breast cancer cases. We suggested that intra-tumoral variability should be taken into consideration when reporting biomarker results. We propose that if there is tumor heterogeneity, all components and their corresponding percentages should be listed in the pathology report.

Some studies showed that amplification of HER2 in breast-cancer cells is associated with clinical responsiveness to anthracycline-containing chemotherapy [21–23] such as CAF (cyclophosphamide, adramycine and 5-FU). In our study, one case (shown in Figure 2 & 3) was treated with four cycles of CAF. After chemotherapy, HER status changed from IHC3+ (positive) to IHC1+ (negative) and correspondingly, the molecular subtype changed from a luminal-HER2 to a luminal molecular subtype. We proposed that the HER2 cluster amplified tumor cells (luminal-HER2) were sensitive to the chemotherapeutic drugs and killed by chemotherapy while tumor cells without HER2 amplification survived.

In summary, our data showed a significant decrease in PR and Ki-67 expression in post-NAC tumors when compared to pre-NAC core biopsies. And for the first time, we reported that larger tumors (≥2cm) and lymph node metastatic breast cancers were more frequently to have those biomarker changes. Moreover, we emphasized that clonal heterogeneity should be taken into consideration when interpreting biomarker expression.

## MATERIALS AND METHODS

### Patients

One hundred and seven patients with invasive ductal breast carcinomas diagnosed from 2011 to 2013 at Shandong University Qilu hospital were enrolled into this study. Each patient received at least three cycles of NAC. All tissue slides from core biopsies and surgical excisions were reviewed by two experienced pathologists (MK and YHQ). Axillary lymph node status was collected retrospectively (Supplementary Table S3). Tumor size were measured grossly, and confirmed under microscope. The response to NAC was assessed by two pathologists independently according to MP grading system. This grading system is based on the reduction of tumor; from MP 1 with no reduction in overall cellularity to MP 5 with no residual malignant cells identified [24, 25]. Grade 1 and grade 2 were regarded as chemotherapy-resistant (CR), and grade 3 to grade 5 tumors were classified as chemotherapy-sensitive (CS) group [24]. All protocols follow the ethical guidelines of the Helsinki Declaration and were approved by Shandong University Research Ethics Committee.

### Immunohistochemistry

All the samples were fixed with 10% (volume/volume) neutral-buffered formalin overnight following CAP protocols and then embedded in paraffin. From each specimen, 5 contiguous 4 μm sections were prepared and used for HE staining and IHC of ER, PR, HER2 and Ki-67. Immunostaining was performed using the Roche Benchmark XT automated slide preparation system (Roche Ltd, Switzerland) following the ultra view DAB v3 procedure with appropriate positive and negative controls.

### Fluorescent in situ hybridization (FISH) and assessment of HER2 gene heterogeneity

FISH was conducted for selected tumors using the PathVysion HER2 DNA Probe kit (Abbott-Vysis Lab, Abbott Park, IL, USA) following the manufacturer's protocol. Two pathologists reviewed the HE and HER2 IHC slides, and marked those cases with heterogeneity at morphological level or at protein level (IHC). For cases with positive tumor heterogeneity, at least 3 tissue spots were counted for the heterogeneity assessment by two pathologists. Each spot was counted for at least 20 nuclei according to ASCO/CAP guideline 2013. A tumor was considered homogeneous if all counted tissue spots showed identical results, while others were considered HER2 gene heterogeneous.

### Scoring methods

The IHC of ER and PR was evaluated independently by two pathologists (MK and YHQ) in a blind manner. 3–5 non-overlapping fields (400 ×) per biopsy were examined. For reproducibility, all of the cores were evaluated twice to confirm the consistency of the results. ER and PR status was assessed by H-score scoring method which takes both the intensity and proportion of positive cells into consideration. The intensity of staining was scored and graded on a 0–3 scale, with no staining = 0, weak staining = 1+, moderate staining = 2+ and strong staining = 3+. The H-score provides an overall score (0–300) based on the sum of positive percentiles of tumor cells. The formula of calculating H-score is: ((1 × % +) + (2 × % ++) + (3 × % +++)). In parallel, according to the ASCO and CAP guidelines, ≥ 1% positive tumor cells were counted as ER or PR positive.

HER2 scoring was performed according to HercepTest criteria with negative (0 and 1+), equivocal (2+), and positive (3+) [25]. Tumors were considered HER2 positive if immunostaining was scored as 3+. HER2/CEP17 ratio more than 2.0 or HER2/per cell more than 6 was also considered HER2 positive. For the amplification cases, those with HER2/CEP17 ≥ 4.0 or HER2 ≥ 10.0 copies per nucleus were regarded as high-level amplification. Cases with HER2/CEP17, 2.0–4.0 or HER2, 6–10 copies per nucleus were regarded as low-level amplification [26, 27]. The cut-off point of 14% for Ki67-positive cells was used to distinguish low proliferation and high proliferation tumors.

All the tumors were classified according to the biomarker status before NAC: luminal A (ER+/PR+/HER2 −/Ki67 < 14%), luminal B (ER+/PR+/HER2 −/Ki67 ≥ 14%), HER2amplified (ER −/PR −/HER2+), triple negative (ER −/PR −/HER2 −), and luminal-HER2 (ER+/PR+/HER2+).

### Statistical methods

Statistical analyses were performed using the SPSS for Windows version 17.0 (SPSS Inc, IL, USA). The Wilcoxon test for paired data was used to compare biomarker changes after NAC. *P*-value < 0.05 was considered statistically significant.

## SUPPLEMENTARY TABLES




