# Supplementary material for: Alterations of biomarker profiles after neoadjuvant chemotherapy in breast cancer: tumor heterogeneity should be taken into consideration
Source: Oncotarget. 2015 Sep 5;6(34):36894–902. doi: 10.18632/oncotarget.5050 (PMC4742218; doi:10.18632/oncotarget.5050)
Supplement: Supplementary file 1 [file oncotarget-06-36894-s001.pdf]

SUPPLEMENTARY TABLES

Supplementary Table S1: ER, PR and HER2 changes between pre-NAC core biopsy and post-NAC excision in molecular subtypes of breast cancer

Molecular subtype		ER (mean ± SE)			PR (mean ± SE)			HER2 (mean ± SE)		
	N	pre-NAC	Post-NAC	*P*	pre-NAC	Post-NAC	*P*	pre-NAC	Post-NAC	*P*
Luminal A	14	176.07 ± 28.81	150.71 ± 31.96	0.541	123.07 ± 30.52	89.64 ± 25.27	0.272	0.36 ± 0.13	0.57 ± 0.23	0.257
Luminal B	38	178.61 ± 16.78	165.76 ± 16.18	0.193	143.84 ± 18.46	103.87 ± 18.14	0.090	0.68 ± 0.13	0.92 ± 0.15	0.115
HER2 amplification	21	0 ± 0.00	5.05 ± 3.34	0.068	0 ± 0.00	0.57 ± 0.34	0.102	2.90 ± 0.07	2.95 ± 0.05	0.564
Triple negative	13	0 ± 0.00	0 ± 0.00	1.000	0 ± 0.00	0 ± 0.00	1.000	0.38 ± 0.14	0.69 ± 0.21	0.102
Luminal-HER2	21	132.86 ± 24.81	95.52 ± 22.80	0.214	66.95 ± 22.01	21.48 ± 7.96	0.073	2.86 ± 0.08	2.52 ± 0.19	0.083

Supplementary Table S2: HER2 status of five breast cancers with discordant reads between biopsies and surgical excisions

Case No.	Pre-NAC	Post-NAC	Comments
QL-12	1+	2+ (HER2/CEP17 = 2.04)	Obvious cell to cell heterogeneity
QL-26	0	2+ (HER2/CEP17 = 2.49)	Three tumor components (morphological)
QL-25	3+	1+	Two tumor components (molecular)
QL-32	2+(HER2/CEP17 = 3.0)	0	Obvious cell to cell heterogeneity
QL-47	2+(HER2/CEP17 = 2.8)	1+	Obvious cell to cell heterogeneity

Supplementary Table S3: Clinicopathological features and biomarker status of 107 breast carcinomas
